# Supplementary material for: A mechanistic model and therapeutic interventions for COVID-19 involving a RAS-mediated bradykinin storm
Source: eLife. 2020 Jul 7;9:e59177. doi: 10.7554/eLife.59177 (PMC7410499; doi:10.7554/eLife.59177)
Supplement: Transparent reporting form [file elife-59177-transrepform.pdf]

eLIFE
Westbrook Centre, Milton Road
Cambridge CB4 1YG
UK
P 01223 855340
W elifesciences.org
T @elife

eLife's transparent reporting form

We encourage authors to provide detailed information *within their submission* to facilitate the interpretation and replication of experiments. Authors can upload supporting documentation to indicate the use of appropriate reporting guidelines for health-related research (see EQUATOR Network), life science research (see the BioSharing Information Resource), or the ARRIVE guidelines for reporting work involving animal research. Where applicable, authors should refer to any relevant reporting standards documents in this form.

If you have any questions, please consult our Journal Policies and/or contact us: editorial@elifesciences.org.

Sample-size estimation

- You should state whether an appropriate sample size was computed when the study was being designed
- You should state the statistical method of sample size computation and any required assumptions
- If no explicit power analysis was used, you should describe how you decided what sample (replicate) size (number) to use

Please outline where this information can be found within the submission (e.g., sections or figure legends), or explain why this information doesn't apply to your submission:

Our analysis is focused on the molecular basis of COVID-19 disease caused by the SARS-CoV-2 coronavirus. We realized early in the pandemic that the RNA-Seq experiments that were used to initially sequence the SARS-CoV-2 virus contained unique and invaluable host information that could explain the pathology of the disease. However, our case sample size was limited to those that were available. In order to make the most accurate comparison, we identified control samples that where of the same type of biological sample and taken in the same manner. We used all control samples.

Replicates

- You should report how often each experiment was performed
- You should include a definition of biological versus technical replication
- The data obtained should be provided and sufficient information should be provided to indicate the number of independent biological and/or technical replicates
- If you encountered any outliers, you should describe how these were handled
- Criteria for exclusion/inclusion of data should be clearly stated
- High-throughput sequence data should be uploaded before submission, with a private link for reviewers provided (these are available from both GEO and ArrayExpress)

Please outline where this information can be found within the submission (e.g., sections or figure legends), or explain why this information doesn't apply to your submission:

eLife Sciences Publications, Ltd is a limited liability non-profit non-stock corporation incorporated in the State of Delaware, USA, with company number 5030732, and is registered in the UK with company number FC030576 and branch number BR015634 at the address Westbrook Centre, Milton Road Cambridge CB4 1YG, UK | March 2019

The COVID-19 BAL samples included 5 biological replicates and four of those 5 had technical replicates. For the control BAL samples, there were 40 biological replicates that included subcategories of smoking, asthma, and obesity. Because this information was unavailable for the COVID-19 samples, we included all of the controls in order be conservative. We ran a PCA analysis using the gene expression values for the controls (see methods) and removed one outlier, leaving 39 control samples. Meta data for all samples is available as supplementary tables.

Statistical reporting

- Statistical analysis methods should be described and justified
- Raw data should be presented in figures whenever informative to do so (typically when N per group is less than 10)
- For each experiment, you should identify the statistical tests used, exact values of N, definitions of center, methods of multiple test correction, and dispersion and precision measures (e.g., mean, median, SD, SEM, confidence intervals; and, for the major substantive results, a measure of effect size (e.g., Pearson's r, Cohen's d)
- Report exact p-values wherever possible alongside the summary statistics and 95% confidence intervals. These should be reported for all key questions and not only when the p-value is less than 0.05.

Please outline where this information can be found within the submission (e.g., sections or figure legends), or explain why this information doesn't apply to your submission:

Statistical approaches can be found in the Methods section and we have included supplementary tables with raw data for transparency. After generating TPM counts for the transcripts from the latest transcriptome (GRCh38_latest_rna.fna) we performed an outlier analysis using PCA and removed one sample from the controls. Differential expression was determined using standard parameters in the *edgeR* package and we adjusted for multiple comparisons. Importantly, we manually inspected read-mappings of differentially expressed genes to ensure that it was not an artifact such as an Alu element. Those which had Alu elements within the mappings were removed from further analyses.

(For large datasets, or papers with a very large number of statistical tests, you may upload a single table file with tests, Ns, etc., with reference to sections in the manuscript.)

Group allocation

- Indicate how samples were allocated into experimental groups (in the case of clinical studies, please specify allocation to treatment method); if randomization was used, please also state if restricted randomization was applied
- Indicate if masking was used during group allocation, data collection and/or data analysis

Please outline where this information can be found within the submission (e.g., sections or figure legends), or explain why this information doesn't apply to your submission:

Identification of cases and controls is provided in Methods and Table S1. They are not ambiguous.

Additional data files ("source data")

- We encourage you to upload relevant additional data files, such as numerical data that are represented as a graph in a figure, or as a summary table
- Where provided, these should be in the most useful format, and they can be uploaded as "Source data" files linked to a main figure or table
- Include model definition files including the full list of parameters used
- Include code used for data analysis (e.g., R, MatLab)
- Avoid stating that data files are "available upon request"

Westbrook Centre, Milton Road
Cambridge CB4 1YG
UK

P 01223 855340
W elifesciences.org
T @elife

Please indicate the figures or tables for which source data files have been provided:

eLife Sciences Publications, Ltd is a limited liability non-profit non-stock corporation incorporated in the State of Delaware, USA, with company number 5030732, and is registered in the UK with company number FC030576 and branch number BR015634 at the address Westbrook Centre, Milton Road Cambridge CB4 1YG, UK | March 2019
